# Psychometric properties of the Adelaide Diagnostic Learning Inventory-Brief (ADLIB)

**DOI:** 10.1186/s13104-020-4884-x

**Published:** 2020-01-15

**Authors:** Harry Minas

**Affiliations:** Global and Cultural Mental Health Unit, Melbourne School of Population and Global Health, The University of Melbourne, Parkville, VIC 3010 Australia

**Keywords:** University students, Learning styles, Adelaide Diagnostic Learning Inventory, Psychometric properties

## Abstract

**Objective:**

There has been increased attention in recent years to mental health, quality of life, stress and academic performance among university students, and the possible influence of learning styles. Brief reliable questionnaires are useful in large-scale multivariate research designs, such as the largely survey-based research on well-being and academic performance of university students. The objective of this study was to examine the psychometric properties of a briefer version of the 39-item Adelaide Diagnostic Learning Inventory.

**Results:**

In two survey samples—medical and physiotherapy students—a 21-item version Adelaide Diagnostic Learning Inventory-Brief (ADLIB) was shown to have the same component structure as the parent instrument, and the component structure of the brief instrument was found to generalise across students of medicine and physiotherapy. Subscale reliability estimations were in the order of magnitude of the parent instrument. Subscale inter-correlations, inter-component congruence coefficients, and correlations between ADLIB subscale scores and several external measures provide support support for the construct and criterion validity of the instrument.

## Introduction

Interest in mental health, quality of life, stress and academic performance among university students, and the possible influence of learning styles, has grown considerably in recent years [1, 2] and there has been a proliferation of instruments designed to measure learning styles and environments. The Adelaide Diagnostic Learning Inventory was one of the earliest of these [3]. Much of the work in this area is survey-based. Brief reliable questionnaires that are applicable across a wide diversity of students are increasingly necessary in multivariate research survey designs, not least because briefer surveys are more likely to be completed by students.

Previous studies have confirmed the reliability and validity of the Adelaide Diagnostic Learning Inventory [3, 4] which is composed of 39 items and measures three dimensions: (1) ‘*Distracted Learning*’—disorganised time management around studying, distraction by social and extra-curricular activities, and low enthusiasm for studying; (2) ‘*Unsuccessful Learning*’—poor organisation of the content to be learned, excessive and ineffective effort expended in studying, and worry about poor performance; and (3) ‘*Successful Learning*’—efforts to integrate ideas, relate ideas to evidence, and questioning or testing ideas against clinical instances or other evidence.

Klimidis et al. [4] showed that the component solution for the ADLI had a strong correspondence with that found by Welch et al. [3] with congruence coefficients (a measure of similarity between two patterns of loadings) ranging between .89 and .92, Cronbach’s α values ranging between .78 and .87 [4].

The objective of this study was to examine the psychometric properties of a briefer 21-item version of the original 39-item version of the ADLI, the Adelaide Diagnostic Learning Inventory-Brief (ADLIB).

## Main text

### Methods

The methods used to reduce the number of items in the instrument sought to avoid the common “sins of short-form development” [5].

The subjects for the present analyses were drawn from two surveys. In the first survey (Sample 1) subjects were 129 fourth year medical students at the University of Melbourne who completed the original 39-item ADLI [4]. In the second survey 200 Physiotherapy students at the University of Melbourne, drawn from all four years of the course, completed the ADLIB (Sample 2). Participants in both samples [5] provided demographic data and completed a range of other instruments (see below). The data from both samples were collected in 1996.

Item selection for retention in the ADLIB was based on:

1. high loadings in the two previously published principal components analyses [3, 4] of the original 39-item ADLI;
2. ensuring that the reduced number of items covered as much of the range as possible in each of the original instrument’s scales; and
3. items that would be applicable to a wide range of student groups, including those outside medical and health sciences fields of study.

Items chosen for removal were those that were minor rewordings of other items with little semantic divergence, and items specifically relevant to medical and health sciences students.

The ADLIB items and the response scale are shown in Additional file 1: Table S1.

Additional measures included in the surveys were the 10-item Patient Interaction Questionnaire (PIQ), the 25-item Medical Practices Anxieties Questionnaire (MPAQ), the 27-item Course Difficulties Inventory (CDI) and the 8-item Brief Measure of English Proficiency (BMEP). Each of these instruments formed a unidimensional scale, with Cronbach’s α values of .77, .92, .94 and .95, respectively. (Details of these instruments may be found in Additional file 1: Tables S2A–E.)

Similar sampling procedures were used for both surveys. Questionnaires were distributed through lecturer and tutor contact with students in class settings and students were asked to return completed questionnaires within a week. The voluntary nature of participation was communicated verbally and in writing at the time of questionnaire distribution and written informed consent was obtained from participants.

### Results

Response rate was 92.8% in the medical students (MS) and 63.0% in the physiotherapy students (PS). Mean age: MS 21.7 years; PS 19.9 years. Male: MS 58.1%; PS 36.0%. Single’ marital status: MS 97.0%; PS 97.5%.

Data from the two samples were analysed using the same procedures. The correlation matrices for the ADLIB items were subjected to principal components analyses. Scree tests in each analysis suggested three main components could be extracted. The three-dimensional solution was rotated using the varimax (orthogonal) method to help label the components, and because it was the method used in the study of the original 39-item ADLI [3, 4]. The final solutions are shown in Table 1 which contains abbreviations of the items (numbered as shown in Additional file 1: Table S1) along with item communality statistics and item variance explained by the dimensions. Loadings of .30 and above are shown in bold type. Overall the three-dimensional solutions account for 51.8% of the item variance in Sample 1 and 48.1% in Sample 2. Only two items did not load uniquely, item 1 in Sample 2 and item 15 in Sample 1. For both samples component 1 corresponds to *Distracted Learning* in previous reports [4, 6] consisting of high-loading items reflecting limited time spent studying, deferring work to the end of term, cramming for exams and preference for social activities rather than work. Component 2, identical with the *Unsuccessful Learning* scale in previous reports, consists of items reflecting a high level of effort in studying, difficulty with organising the content of learning and dissatisfaction with or concern about performance outcomes. Component 3 corresponds to the *Successful Learning* component in the original ADLI analyses, consisting of high-loading items that reflect efforts to integrate ideas and information into a broader framework, expenditure of cognitive effort on ideas and information, and testing interpretations of results.

**Table 1:** Varimax rotated factor solutions of the ADLIB for two samples

Items	Sample 1 4th year medical students (n = 129)				Sample 2 physiotherapy students (n = 200)			
	Comp 1	Comp 2	Comp 3	Communal	Comp 1	Comp 2	Comp 3	Communal
Distracted Learning (DSL)								
16. Don’t spent enough time studying	.84	.09	− .10	.71	.81	− .14	− .05	.67
18. Impossible to keep a regular schedule	.83	.22	.00	.73	.68	.07	− .09	.48
1. Spend less time studying than others	.80	− .02	.07	.64	.73	− .30	− .10	.64
3. Put off work to end of term	.80	.03	− .01	.65	.77	.10	.02	.60
4. More interested in social life than work	.77	.09	− .12	.62	.57	− .04	− .10	.34
20. Cope with exams by cramming	.62	.05	− .18	.42	.72	.05	− .03	.52
17. No time to look over notes during term time	.53	.01	− .08	.29	.62	.03	− .21	.43
Anxious/Inefficient Learning (AIL)								
10. Much effort in learning but it doesn’t stick	− .21	.79	− .08	.67	− .25	.77	.11	.67
12. Work hard but rarely satisfied with results	− .15	.77	− .07	.63	− .17	.67	.10	.48
7. worry About failing exams	.11	.69	.03	.49	.04	.64	− .09	.42
2. Concentrate on detail and lose big picture	.22	.67	− .12	.52	− .05	.60	.00	.36
11. Worry about coping with course	.11	.61	− .06	.39	.00	.72	.06	.53
19. Difficulty fitting facts into a big picture	.10	.62	− .04	.40	.08	.61	− .25	.44
15. Trouble organising information	.36	.57	-.16	.48	.24	.67	-.15	.53
Independent/Deep Learning (IDL)								
14. Visualise situation learnt material might apply	− .03	.07	.74	.55	− .16	− .09	.69	.52
6. Play with own ideas about studied material	− .07	− .13	.71	.52	.10	− .15	.75	.59
9. Map out new topic for myself	.04	.06	.65	.43	− .14	.11	.51	.29
8. Relate new ideas to real-life experience	.04	− .12	.64	.42	− .06	− .10	.69	.49
21. Try to work out alternative interpretation of results	− .20	− .03	.63	.44	− .02	− .01	.54	.29
5. Try to think of alternative solutions and test them	− .09	− .10	.65	.44	− .02	− .06	.63	.41
13. After reading something important, I think about it	− .17	− .23	.60	.44	− .21	.00	.59	.39
				Total				Total
Percent variance	24.3	15.1	12.5	51.8	20.1	17.0	11.0	48.1

In this report the Component 1 title, *Distracted Learning* (DSL), has been retained. The titles of Components 2 and 3 have been changed from *Unsuccessful Learning* to *Anxious/Inefficient Learning* (AIL) for Component 2, and from *Successful Learning* to *Independent/Deep Learning* (IDL) for Component 3, because the original titles imply learning outcomes rather than referring to learning styles.

Internal consistency reliability among the items composing each component of the ADLIB was calculated using the Cronbach’s α statistic. Table 2 shows the α coefficients along the diagonal of the matrix (bold text). All values were between .75 and .87, and were comparable to the long version of the corresponding scales [4]. In Sample 1, which completed the 39-item ADLI, correlations between scales DSL, AIL and IDL from the ADLIB and corresponding scales from the ADLI were .95, .96 and .89, respectively, demonstrating that abbreviating the scales resulted in little loss of information.

**Table 2:** Cronbach’s alpha coefficients (bold), inter-correlations (italics), and inter-factor congruence coefficients (underlined) for the two samples

	Sample 1: medical students			Sample 2: physiotherapy students		
	DSL	AIL	IDL	DSL	AIL	IDL
DSL	**.87**	–	–	–	–	–
AIL	.20*	**.81**	–	–	–	–
IDL	− .19*	− .21*	.**79**	–	–	–
DSL	*.96*	*.06*	*− .18*	**.84**	–	–
AIL	*.04*	*.96*	*− .17*	*− .05*	**.80**	–
IDL	*− .22*	*− .14*	*.96*	− .19**	*− .11*	**.75**

*DSL* Distracted Learning scale, *AIL* Anxious/Inefficient Learning scale, *IDL* Independent/Deep Learning Scale

* p < .05; ** p < .01

The similarity in component structure across the two samples was explored using Harman’s [7] congruence coefficients, which indicate the degree of similarity in item loadings among the component matrices. Table 2 shows a summary of congruence coefficients (shown in plain text). Underlined in the box are the congruence coefficients for corresponding components in the two samples—these values were all .96. Coefficients for non-corresponding components were low in value, as would be expected if the overall solutions were similar. These independent measures across two samples, one medical and the other physiotherapy students, is further evidence for measurement invariance (configural) of the instrument. The instrument is generalisable across groups.

Table 2 also shows correlations between the ADLIB subscale scores across the two samples. Examination of the correlation matrices (shown in italic typescript in Table 2) across the two samples shows there was a significant negative correlation between DSL and IDL scores. That is, compared with low DSL scorers, high DSL scorers (i.e., students who endorse items reflecting poor time management, preference for socialisation over study, etc.) tend not to use learning strategies that involve effort to cognitively organise and contextualise information. Additionally, in the medical student sample, there was a significant positive correlation between DSL and AIL scores. High DSL scorers, compared to low scorers, tend to report more difficulties with managing course information and to report more worry about academic performance. In the medical student sample there was also a significant negative correlation between IDL and AIL scores. That is, those tending to report use of IDL strategies, relative to low scorers on the IDL scale, were less likely to endorse items from the AIL scale, such as worrying about performance, expending effort in unproductive study and having trouble organising information. Scale scores are measuring different latent constructs, reflecting the learning styles suggested by the scale labels.

Table 3 shows the correlations of the ADLIB subscales with relevant external measures, contributing to establishment of concurrent criterion validity of the scales. DSL scores are not correlated with scores any of the four external measures. AIL is correlated significantly and in the expected directions with all four external measures. High scorers on the AIL scale were more likely to have lower scores on the Patient Interaction Questionnaire (indicating lower confidence in their interactions with patients), higher scores on the Medical Practices Anxiety Questionnaire, higher scores on the Course Difficulties Inventory and lower scores on the Brief Measure of English Proficiency. Scores on the IDL scale are correlated significantly (and negatively) only with scores on the Course Difficulties Inventory.

**Table 3:** Correlations between the three ADLIB sub-scale scores and other measures

	DSL	AIL	IDL
Sample 1: medical students			
Patient Interaction Questionnaire (PIQ) (n = 129)	.02	− .37***	.15
Medical Practices Anxieties Questionnaire (MPAQ) (n = 129)	.01	.20*	− .10
Sample 2: physiotherapy students			
Course Difficulties Inventory (CDI) (n = 200)	.00	.50***	− .24**
Brief Measure of English Proficiency (BMEP) (n = 47)	.05	− .40***	− .14

Sample sizes vary according to missing values or, in the case of English Language Proficiency, the completion of the scale only by overseas-born students

*DSL* Distracted Learning scale, *AIL* Anxious/Inefficient Learning scale, *IDL* Independent/Deep Learning scale

* p < .05; ** p < .01; *** p < .001

### Discussion

This study reports psychometric properties of the Adelaide Diagnostic Learning Inventory-Brief (ADLIB), a 21-item version of the 39-item Adelaide Diagnostic Learning Inventory.

The three-dimensional structure of the ADLIB is replicated across the two samples studied, and the item composition of the three components was the same as that in the previous studies of the 39-item version [3, 4], indicating configural invariance of the instrument. The first dimension of the ADLIB, *Distracted Learning* (DSL), consists of items reflecting poor time management and distraction from studying, diminished commitment to studying, and a preference for social activities over studying. The second dimension, Anxious/Inefficient Learning (AIL), contains two highly related sets of items, the first reflecting extensive but ineffective expenditure of effort in studying and anxiety about poor performance, and the second reflecting difficulty in organising information into a general framework. The third dimension, Independent/Deep Learning (IDL), consists of items reflecting systematic and independent attempts by students to organise information, to link it to relevant contexts, to work on ideas, to question them and to seek alternative understandings and explanations.

Scores from each of the ADLIB subscales are correlated in a coherent and largely predictable manner with several relevant external measures, suggesting adequate construct validity. In particular, the AIL scale is correlated with all the external measures used in the surveys, suggesting the possible value of further exploration of the AIL scale as a predictor of low confidence with patient interaction, anxieties about conducting medical procedures, and likelihood of experiencing difficulties in coping with the course of study. In addition, the results suggest that immigrant students with lower levels of English proficiency may be more likely to score high on AIL and may be more likely to experience the problems measured in the PIQ, MPAQ and CDI scales. Given the content of the AIL scale it may be reasonable to hypothesise that a high AIL score may be associated with, and may predict, poorer academic performance, i.e. that the AIL scale may have predictive validity and may be useful in early identification of students likely to get into subsequent difficulties in academic performance. This should be tested in a sufficiently large and diverse sample of students where objective measures of academic performance are available.

### Conclusions

This study suggests that ADLIB may be a robust measure of learning styles in health sciences students, although the validity of the scales needs to be further tested against a broader range of external correlates, particularly measures indicative of educational outcomes of interest, such as academic performance.

Given that all except one of the items (item 21: “In looking at experimental or clinical results, I generally try to work out several alternative interpretations”) chosen for inclusion in the ADLIB were intentionally selected because they are not specific to health discipline students it would be useful to test the reliability and validity of a 20-item version of the instrument (with item 21 excluded) in surveys of broader samples of students from a wide range of academic disciplines.

## Limitations

- Small sample sizes.
- Use of self-report measures rather than independent criterion measures.
- Cross-sectional study precluding any causal inferences.

## Supplementary information

**Additional file 1: Table S1.** Adelaide Diagnostic Learning Inventory—Brief (ADLIB). **Table S2A.** Measures included in one or both surveys. **Table S2B.** Patient Interaction Questionnaire (PIQ). **Table S2C.** Medical Practices Anxieties Questionnaire (MPAQ). **Table S2D.** Course Difficulties Inventory (CDI). **Table S2E.** Brief Measure of English Proficiency (BMEP).

## Data Availability

Additional data and other details in Additional file.

## Abbreviations

ADLI: Adelaide Diagnostic Learning Inventory
ADLIB: Adelaide Diagnostic Learning Inventory-Brief
AIL: Anxious/Inefficient Learning scale
BMEP: Brief Measure of English Proficiency
CDI: Course Difficulties Inventory
DSL: Distracted Learning scale
IDL: Independent/Deep Learning scale
MPAQ: Medical Practices Anxieties Questionnaire
MS: medical students
PIQ: Patient Interaction Questionnaire
PS: physiotherapy students
