# Supplementary material for: Psychometric properties of the Adelaide Diagnostic Learning Inventory-Brief (ADLIB)
Source: BMC Res Notes. 2020 Jan 15;13:31. doi: 10.1186/s13104-020-4884-x (PMC6964103; doi:10.1186/s13104-020-4884-x)
Supplement: Supplementary file 1 — Additional file 1: Table S1. Adelaide Diagnostic Learning Inventory—Brief (ADLIB). Table S2A. Measures included in one or both surveys. Table S2B. Patient Interaction Questionnaire (PIQ). Table S2C. Medical Practices Anxieties Questionnaire (MPAQ). Table S2D. Course Difficulties Inventory (CDI). Table S2E. Brief Measure of English Proficiency (BMEP). [file 13104_2020_4884_MOESM1_ESM.pdf]

Psychometric properties of the Adelaide Diagnostic Learning Inventory - Brief (ADLIB): Additional File

Harry Minas

Additional file 1: Supplementary Table S1: Adelaide Diagnostic Learning Inventory – Brief (ADLIB)

Item	Response
1. I seem to spend less time studying than most students.	Please indicate your level of agreement with each of the following statements:<br><br>0 Strongly disagree<br>1 Partly disagree<br>2 Partly agree<br>3 Strongly agree
2. I concentrate on the detail so much that I seem to lose sight of the general picture.	
3. My habit of putting off work leaves me with far too much to do at the end of term.	
4. I suppose I am more interested in the social life than the academic work.	
5. When confronted with a difficult or unsolved problem, I often try to think up possible solutions and then check them out.	
6. I like to play around with ideas of my own related to what I'm studying.	
7. I worry a lot about whether I will fail the exams.	
8. In trying to understand new ideas I generally set out to relate them, if possible, to real-life experience.	
9. I find it helpful to 'map out' a new topic for myself seeing how the ideas fit together.	
10. I generally put a lot of effort into learning, but I still have trouble making it stick.	
11. I often worry about whether I will be able to cope with this course itself.	
12. I work hard but am rarely satisfied with my results.	
13. After reading something important, I often think about it for a while.	
14. When I am tackling something new, I often try to visualise a situation to which it might apply.	
15. I have trouble organising the information I have to remember.	
16. I suppose I really do not spend enough time studying.	
17. I rarely find time to look over my notes during term-time.	
18. I find it impossible to keep a regular schedule for studying.	
19. Although I generally remember facts and details, I find it difficult to fit them together into an overall picture.	
20. I usually cope with exams by 'cramming' just before they come up.	
21. In looking at experimental or clinical results, I generally try to work out several alternative interpretations.	

Additional file 1: Supplementary Table S2A: Measures included in one or both surveys

Measure	Items	Response	Components	Conbach's α
**Adelaide Diagnostic Learning Inventory-Brief (**ADLIB)<br>Included in Sample 1: medical students and Sample 2: physiotherapy students survey<br>Additional File 1	21	0 Strongly disagree<br>1 Partly disagree<br>2 Partly agree<br>3 Strongly agree	3	Range:<br>0.75 – 0.87
**Patient Interaction Questionnaire** (PIQ)<br>Included in Sample 1: medical students survey<br>Additional File 2	10	0 Strongly disagree<br>1 Partly disagree<br>2 Partly agree<br>3 Strongly agree	1	0.77
**Medical Practices Anxieties Questionnaire** (MPAQ)<br>Included in Sample 1: medical students survey<br>Additional File 3	25	0 Not anxious<br>1 Slightly anxious<br>2 Fairly anxious<br>3 Very anxious	1	0.92
**Course Difficulties Inventory** (CDI)<br>Included in Sample 2: physiotherapy students survey<br>Additional File 4	27	0 Not at all difficult<br>1 A little difficult<br>2 Quite difficult<br>3 Very difficult	1	0.94
**Brief Measure of English Proficiency** (BMEP)<br>Included in Sample 2: physiotherapy students survey<br>Additional File 5	8	0 Poor<br>1 Fair<br>2 Good<br>3 Very good<br>4 Excellent	1	0.95

Additional file 1: Supplementary Table S2B: Patient Interaction Questionnaire (PIQ)

(Adapted from: Orbell, S. & Abraham, C. (1993) Behavioural sciences and the real world: report of a community interview scheme for medical student. *Medical Education*, 27:218-228.)

Item	Response
1. I am very confident about interviewing patients.	Please indicate your level of agreement with each of the following statements:<br><br>0 Strongly disagree<br>1 Partly disagree<br>2 Partly agree<br>3 Strongly agree
2. I think patients are, in general, very willing to discuss their medical and personal problems with medical students.	
3. In general, I think patients benefit from being interviewed and examined by medical students.	
4. I think I am quite skillful at asking patients questions which will elicit all the necessary information.	
5. I think I am quite good at expressing to patients my understanding of their feelings during interview.	
6. I think I am quite good at communicating to patients my interest in patients' beliefs and experiences.	
7. I concentrate very well on what patients are saying when I interview them.	
8. I am quite good at making notes during a patient interview without disrupting the flow of the interview.	
9. I think I am very aware of the importance of social and psychological factors in patients' health.	
10. I get very anxious before interviewing patients.	

Unidimensional scale. Conbach's α = 0.77

Additional file 1: Supplementary Table S2C: Medical Practices Anxieties Questionnaire (MPAQ)

(Adapted from: Moss, F. & McManus, C. (1992) The anxieties of new clinical students. *Medical Education*, 26:17-30)

Item	Response
1. Getting diagnoses wrong	Please indicate how anxious each of the following situations makes you feel:
2. Presenting cases on ward rounds	
3. Inadvertently hurting patients	
4. Helping with a cardiac arrest	
5. Telling consultants that you do not know something	0 Not anxious
6. Suturing patients in casualty	1 Slightly anxious
7. Dealing with drunk/abusive patients	2 Fairly anxious
8. Getting infected by patients	3 Very anxious
9. Dealing with psychiatric patients	
10. Making diagnoses	
11. Delivering babies	
12. Talking with dying patients	
13. Carrying out rectal examinations	
14. Carrying out vaginal examinations	
15. Being asked difficult questions by patients	
16. Dealing with sick children	
17. Talking to seriously ill patients	
18. Explaining to a patient that a diagnosis is not known	
19. Taking blood from patients	
20. Giving injections	
21. Examining patients	
22. Going to post-mortems	
23. Being up all night	
24. Going to operating theatre	
25. Undressing patients of the opposite sex	

Unidimensional scale. Conbach's α = 0.92

Additional file 1: Supplementary Table S2D: Course Difficulties Inventory (CDI)

Item	Response
1. understanding what lecturers or tutors say	Please indicate how difficult you have found the following tasks and situations. If you have not been faced with a particular situation please answer "not applicable".
2. taking useful notes in lectures	
3. using technical equipment (e.g., in laboratory work, computers, etc.)	
4. participating in seminars or tutorials	
5. using the library facilities and services	
6. writing reports, essays, assignments or case reports	
7. doing multiple choice tests	
8. understanding medical textbooks	
9. doing tests requiring written answers	0 Not at all difficult
10. replying verbally to questions by tutors	1 A little difficult
12. making yourself understood by others	2 Quite difficult
13. relating to lecturers and tutors on a social level	3 Very difficult
15. keeping up with required reading	
16. giving verbal presentations in seminars or tutorials	
17. submitting assignments on time	
18. memorising medical information	
19. asking questions in lectures	
20. asking questions in tutorials	
21. asking for help from tutors	
22. expressing an opinion in class	
23. presenting a clinical case to the class	
24. presenting a clinical case to examiners	
25. relating theory and information to practical clinical skills	
26. following instructions in practical laboratory assignments	
27. conducting a clinical examination of a patient on which you will be assessed	

Unidimensional scale. Conbach's $\alpha = 0.94$

Additional file 1: Supplementary Table S2E: Brief Measure of English Proficiency (BMEP)

Item	Response
1. Understanding people in social situations (on the phone, in shops, at parties etc)<br>2. Writing assignments/exams<br>3. Reading academic literature for your course<br>4. Speaking/communicating in social situations<br>5. Reading fiction books, magazines etc<br>6. Writing personal letters, notes, messages<br>7. Understanding what others (tutors, lecturers, other students) are saying in class<br>8. Speaking/communicating in academic situations	How would you rate your English language ability in the following areas?<br><br>0 Poor<br>1 Fair<br>2 Good<br>3 Very good<br>4 Excellent

Unidimensional scale. Conbach's α = 0.95
